# Supplementary figures and images for: The E2F family as potential biomarkers and therapeutic targets in colon cancer
Source: PeerJ. 2020 Feb 19;8:e8562. doi: 10.7717/peerj.8562 (PMC7035869; doi:10.7717/peerj.8562)

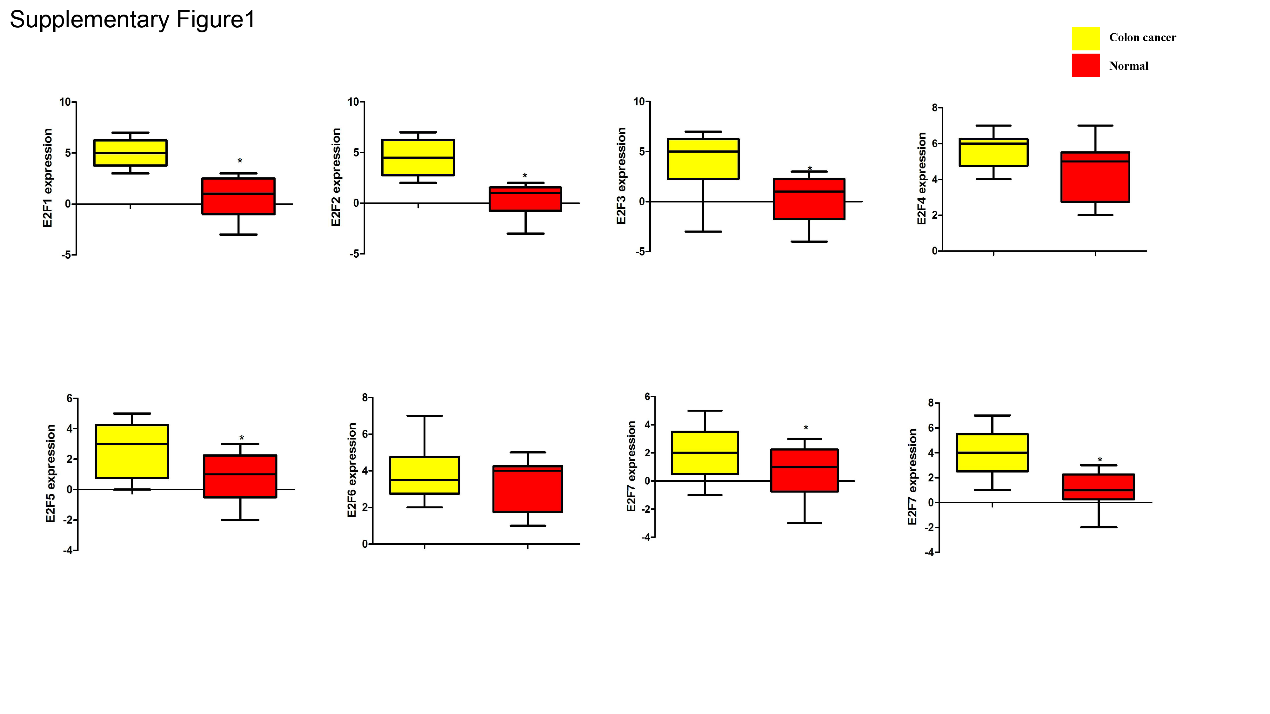

Supplement: Figure S1 [file peerj-08-8562-s003.tif]

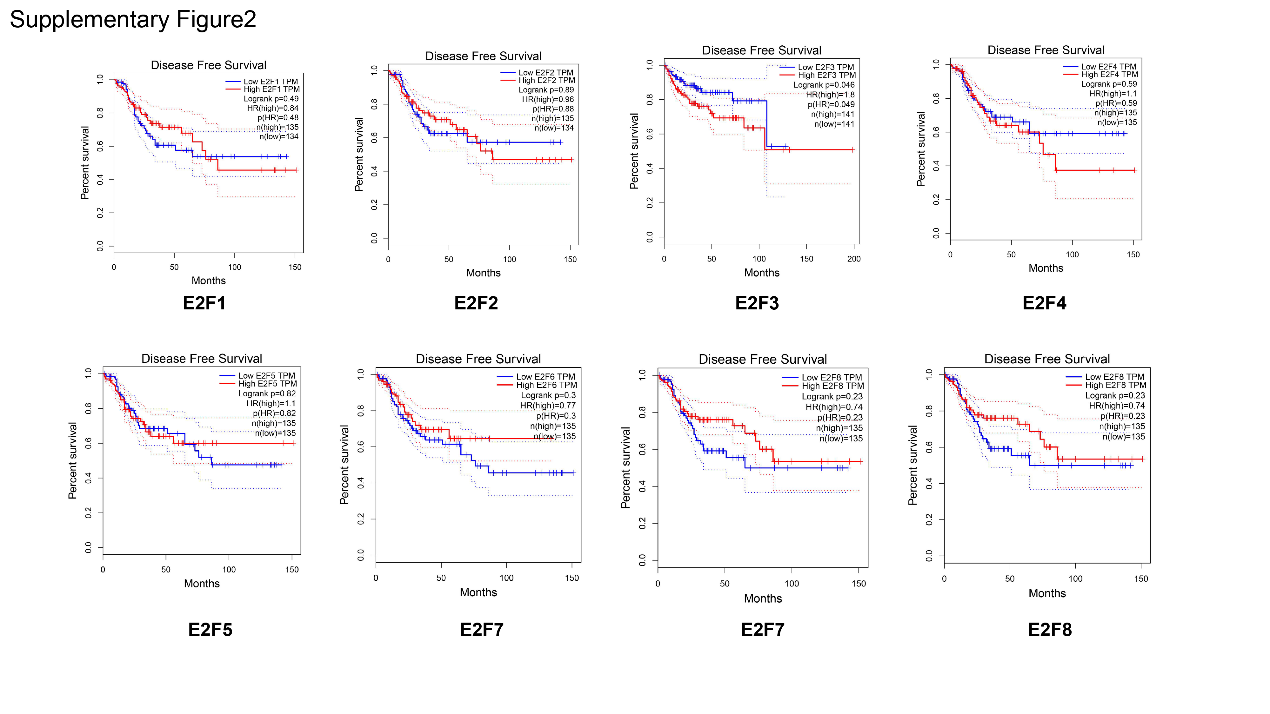

Supplement: Figure S2 [file peerj-08-8562-s004.tif]

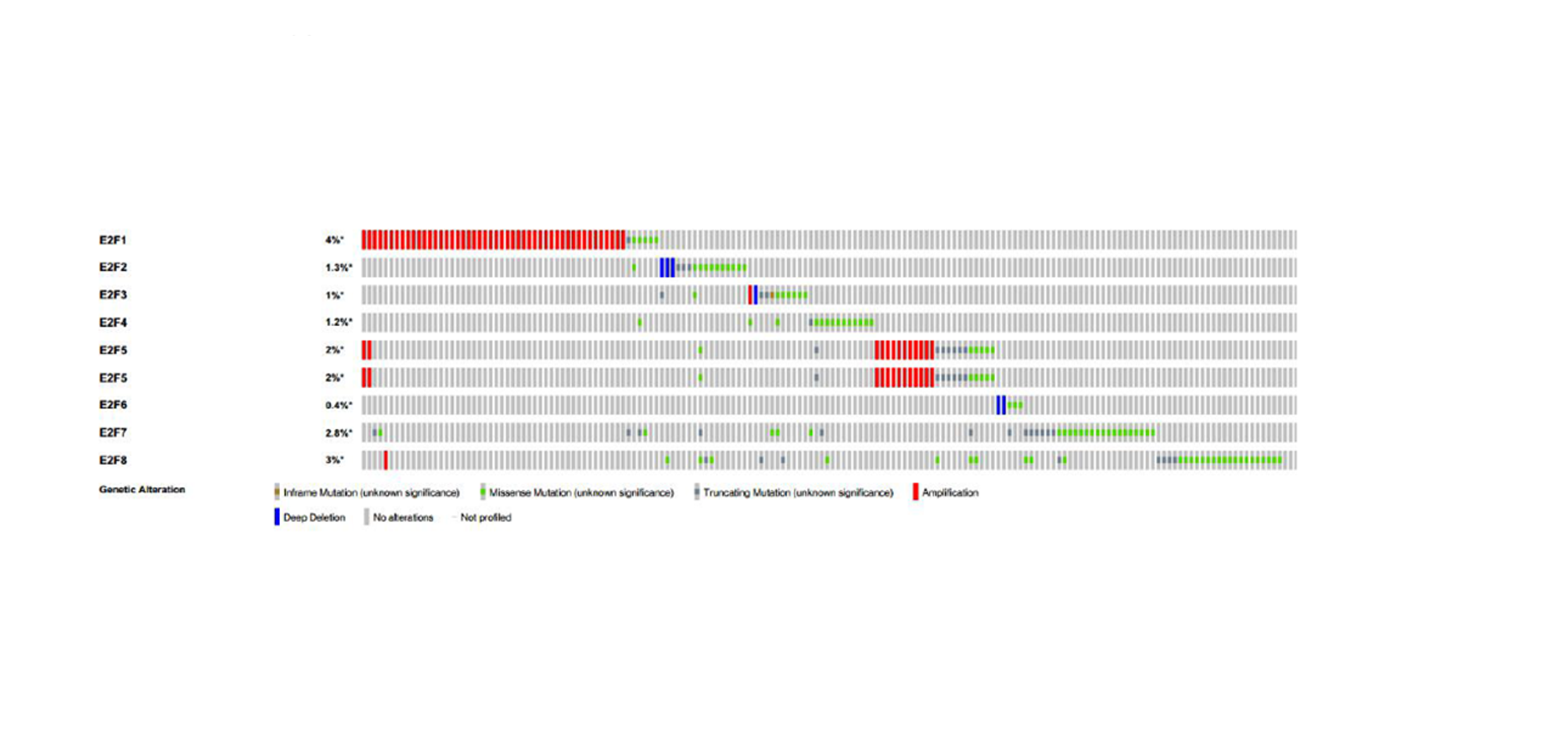

Supplement: Figure S3 [file peerj-08-8562-s005.tif]
