# Supplementary material for: The E2F family as potential biomarkers and therapeutic targets in colon cancer
Source: PeerJ. 2020 Feb 19;8:e8562. doi: 10.7717/peerj.8562 (PMC7035869; doi:10.7717/peerj.8562)
Supplement: Supplemental Information 1 [file peerj-08-8562-s001.docx]

Public database:

GEPIA: <http://gepia.cancer-pku.cn/detail.php>

HPA: <https://www.proteinatlas.org/>

Oncomine database: <https://www.oncomine.org/>

Dear editor,

GEPIA is a newly developed interactive web server for analyzing the RNA sequencing expression data of 9,736 tumors and 8,587 normal samples based on the TCGA and the GTEx projects, using a standard processing pipeline. Our study is not based on one or two chips, it is calculated from the whole database of GEPIA.

Similar to GEPIA, HPA is also dynamically changing database which may be updated everyday. So we cannot provide the chip number as you mentioned. They may be totally different.

Best regards.
